# Multipotent Mesenchymal Stem Cell Therapy for Vascular Dementia

**DOI:** 10.3390/cells14090651

**Published:** 2025-04-29

**Authors:** Eun-Young Kim, Ki-Sung Hong, Dong-Hun Lee, Eun Chae Lee, Hyung-Min Chung, Se-Pill Park, Man Ryul Lee, Jae Sang Oh

**Affiliations:** 1 Miraecellbio Co., Ltd., Seoul 04795, Republic of Korea; jlokey@miraecellbio.com (E.-Y.K.); kshong@miraecellbio.com (K.-S.H.); hmchung@kku.ac.kr (H.-M.C.); sppark@jejunu.ac.kr (S.-P.P.); 2 Industry-Academic Cooperation Foundation, The Catholic University of Korea, 222 Banpo-daro, Seocho-gu, Seoul 06591, Republic of Korea; madeby58@gmail.com; 3 Department of Medical Life Sciences, College of Medicine, The Catholic University of Korea, 222 Banpo-daero, Seocho-gu, Seoul 06591, Republic of Korea; lec9589@gmail.com; 4 Department of Medical Sciences, College of Medicine, The Catholic University of Korea, 222 Banpo-daero, Seocho-gu, Seoul 06591, Republic of Korea; 5 Department of Stem Cell Biology, School of Medicine, Konkuk University, 268 Chungwon-daero, Chungju 27478, Republic of Korea; 6 Faculty of Biotechnology, College of Applied Life Sciences, Jeju National University, Jeju 63243, Republic of Korea; 7 Department of Stem Cell and Regenerative Biotechnology, KU Institute of Science and Technology, Konkuk University, 120 Neungdong-ro Gwangjin-gu, Seoul 05029, Republic of Korea; 8 Department of Neurosurgery, Uijeongbu St. Mary’s Hospital, College of Medicine, The Catholic University of Korea, Seoul 06591, Republic of Korea

**Keywords:** vascular dementia, stem cells, human embryonic stem cells, carotid stenosis, cognitive dysfunction, conduct disorder

## Abstract

Vascular dementia (VD), characterized by cognitive decline and behavioral disorders, has seen a rapid increase in prevalence in recent years. However, effective treatments for VD remain unavailable. Due to its regenerative potential, stem cell therapy has garnered attention as a promising approach for VD treatment, yet it has shown limited effects on cognitive and behavioral impairments caused by the disease. To address this limitation, this study aimed to develop a novel treatment using human embryonic stem cell-derived multipotent mesenchymal stem cells (MMSCs). The therapeutic efficacy of MMSCs was evaluated using a vascular dementia mouse model induced by bilateral carotid artery stenosis (BCAS). The effects of MMSCs were assessed through behavioral tests and postmortem brain tissue analysis, including mRNA expression analysis and hematoxylin and eosin (H&E) staining. MMSCs treatment significantly improved both working memory and long-term memory. Histological analysis revealed enhanced angiogenesis, preservation of blood–brain barrier integrity, and improved hippocampal organization. Furthermore, MMSCs treatment reduced the expression of *Rock1/2*, indicating suppression of neuroinflammatory and apoptotic pathways. These findings suggest that MMSCs offer a sustainable and effective therapeutic approach for vascular dementia.

## 1. Introduction

Vascular dementia (VD) or vascular cognitive impairment is caused by impaired blood flow to the brain, which reduces the supply of oxygen and nutrients necessary for brain function, resulting in neuronal cell death, inflammation, and inefficient energy metabolism [1]. It is the second most common cause of dementia, following Alzheimer’s disease. The prevalence of VD doubles every 5.3 years with aging, and it is a common form of dementia, accounting for 15–20% of all cases of dementia [2,3]. Despite progress in VD treatment in the past decade, the mechanisms underlying the development of this disorder remain unclear [4]. Thus, effective treatments for VD are still lacking, and many patients still suffer from long-term cognitive and behavioral disorders [5]. To date, an FDA-recommended treatment for VD remains unavailable. Cholinesterase inhibitors and memantine are widely used for clinical treatment of VD; however, these drugs have been approved for the treatment of Alzheimer’s disease and thus are unlikely to be effective for VD alone [6]. In particular, the acetylcholinesterase inhibitor donepezil exerts neuroprotective and anti-inflammatory effects by increasing acetylcholine concentration in neurotransmitters and regulating acetylcholine receptors. However, its therapeutic effect is unexpected in patients with VD because of the intact cholinergic system [7].

Further research and development of treatments for VD is hindered by the lack of molecular standards and preclinical animal models [8]. Although cerebrovascular damage affects cognitive function, significant clinical differences between diverse subtypes and causes of VD complicate the treatment and research of this disorder [9]. Therefore, animal models must be established, and molecular characteristics must be defined to study various forms of VD [10]. In our previous study, we established a VD animal model presenting hypoxia-induced white matter injury by damaging carotid arteries through bilateral common carotid artery stenosis (BCAS) surgery, thus reducing cerebral blood flow [11]. In particular, upregulated expression of Rock 1/2 that regulates the cytoskeleton and downregulated expression of tight junction (TJ) gene in the brain tissue of mice with BCAS-induced VD led to the collapse of the blood–brain barrier (BBB), causing the influx of pro-inflammatory cytokines and the subsequent death of nerve cells [11]. This animal model shows progressive damage to white matter, cortex, and hippocampus, causing behavioral deficits due to downregulation of factors related to walking memory, long-term cognitive function, and locomotor activity [11]. Such preceding research can be used as preclinical research to develop novel treatments for VD.

Drugs that can arrest or slow the progression of VD are currently lacking. Stem cell transplantation is being considered as a method to markedly restore the brain and blood vessels of patients with VD caused by vascular occlusion. Given their self-renewal and differentiation capabilities, stem cells have attracted attention for the treatment of various degenerative diseases [12]. In particular, several clinical studies have used various stem cells for the treatment of degenerative brain diseases such as stroke, but cell therapy has yet to be approved as a treatment for such conditions [13]. Stem cells with proven therapeutic effects are necessary to overcome the limitations of existing cell therapy [14].

In the present study, we aimed to use multipotent mesenchymal stem cells (MMSCs) differentiated from human embryonic stem cells (hESCs) and transplant them into an animal model of VD to evaluate their efficacy. The MMSCs used in this study are superior to adult-derived mesenchymal stem cells (MSCs) in their ability to proliferate and function as immune modulators. Thus, they are expected to help treat cognitive and behavioral disorders caused by VD. This study may serve as a basis for developing hESC-derived cells as a novel treatment for VD.

## 2. Materials and Methods

### 2.1. Experimental Animal Protocols and Study Framework

All experiments were conducted at the Experimental Animal Center of the Soonchunhyang Institute of Medi-Bio Science (SIMS, Cheonan, Republic of Korea) in accordance with the ARRIVE guidelines. Mice were maintained under controlled conditions: a 12-h light–dark cycle (7:00 a.m.–7:00 p.m.), a temperature of 23 ± 1–2 °C, and a humidity level of 50 ± 5%. All animal procedures were approved by the Institutional Animal Care and Use Committee of Soonchunhyang University (IACUC No. SCH 20-0065) and adhered to the Guide for the Care and Use of Laboratory Animals issued by the National Research Council, approval date: 9 November 2020.

### 2.2. Experimental Animals and Group Classification

To perform a preclinical animal model for cell therapy products, female nude mice (*BALB/c-Foxn1 nu/Arc*, 9 weeks old) were purchased from OrientBio (Seongnam, Republic of Korea). After a one-week habituation period, BCAS (bilateral common carotid artery stenosis) surgery was performed at 10 weeks of age to establish a vascular dementia model. After the surgery, there was e a 6-week period of narrowing of the blood vessels. At 16 weeks, mice weighing an average of 18 g were randomly selected and assigned to groups for the experiment: normal type (NT, control group without surgery or treatment, n = 10), vascular dementia (VD, disease group, n = 10), MMSC (MMSCs treatment group, n = 10), BMSC (bone marrow-derived MSCs treatment group, n = 10), and Done (donepezil treatment group, n = 10).

### 2.3. BCAS Surgery

The experimental design was based on a previously reported study and involved micro coil implantation (SWPAO. 080.180.5×2.5, Samini Spring/Sawane, Shizuoka, Japan). Briefly, mice were anesthetized using 2% isoflurane solution (Hana Pharm, Seongnam-si, Republic of Korea) for 8 min, followed by the maintenance of anesthesia with a mixed gas of oxygen and nitrogen dioxide. The mice were placed in a supine position and secured on a microscope platform. A midline cervical incision was made to expose the common carotid arteries, which were carefully isolated from surrounding sheaths. Micro coils were then applied bilaterally to the carotid arteries to induce stenosis, and 6-0 silk sutures were used to secure the coils. All surgical procedures were performed on a heating pad maintained at 25–26 °C to prevent hypothermia. Postoperatively, mice were monitored twice a week for body weight, body temperature, and paralysis until they reached a stable condition.

### 2.4. Cell Culture

Embryonic stem cell lines for the establishment of hESC-MMSCs were maintained in Endothelial Cell Growth Medium MV2 (Cat: C-22022, PromoCell, Heidelberg, Germany) supplemented with penicillin-streptomycin solution (100×, Corning, NY, USA) in tissue culture dishes (100 × 20 mm style, Corning, NY, USA). Cells were dissociated using TrypLE Express (Gibco, Grand Island, NY, USA) for 3 min. For experimental purposes, MMSCs were prepared at a density of 5 × 10^5^ cells and incubated in Eppendorf tubes at 37 °C in a 5% CO_2_ atmosphere. BMSCs were cultured in Dulbecco’s Modified Eagle Medium (DMEM) supplemented with 10% fetal bovine serum (FBS) and 1% penicillin-streptomycin. BMSCs were prepared at a density of 2 × 10^5^ cells and incubated under the same conditions at 37 °C in a 5% CO_2_ atmosphere.

### 2.5. Administration of Medication

Prior to administration, donepezil (Yuhan, Republic of Korea) was pulverized using a mortar and pestle, dissolved in saline, and prepared at a final concentration corresponding to 20 mg/kg body weight. The MMSC and BMSC groups were administered via intracerebroventricular (ICV) injection, while the donepezil group was administered via intraperitoneal (IP) injection.

### 2.6. Cellular Therapy and Stereotaxic Brain Injection

One week after BCAS modeling surgery, cell therapy was performed via stereotaxic surgery. Mice were anesthetized with isoflurane (Hana Pharm, Seongnam-si, Republic of Korea) using respiratory anesthesia, and the surgical coordinates were determined relative to the bregma (M/L 1.1, A/P 0.5, D/V 2.5 mm). The marked area was drilled using a medical-grade drill, and the mice were secured on a stereotaxic apparatus (Harvard Apparatus, Holliston, MA, USA). Before cell administration, 3 µL of cerebrospinal fluid (CSF) was aspirated from the ventricle using a Hamilton syringe (10 µL Gastight Syringe, Model 1701 RN, Small Removable Needle, 26s gauge, 2 in, point style 2, Hamilton, Reno, NV, USA). Each cell suspension was prepared in 3 µL of phosphate-buffered saline (PBS) and injected into the ventricle at the defined coordinates.

### 2.7. Behavioral Tests

All behavioral analyses were conducted at the Experimental Animal Center of SIMS (Cheonan, Republic of Korea) in a custom-designed chamber (domestically manufactured, Republic of Korea) to minimize experimental variability, with white noise maintained at approximately 60 dB. Behavioral assessments were performed using Smart v3.0 (Panlab, Barcelona, Spain) and shut-avoidance software v3.0 (Panlab, Barcelona, Spain). Each experiment was conducted in triplicate, and the light intensity within the chamber was maintained at 390 lux.

#### 2.7.1. Y-Maze Test

Each mouse underwent a Y-maze test to assess working memory. The Y-maze apparatus consisted of three arms (A, B, and C) and a central zone, forming a “Y” shape. The arms were made of opaque material, with each arm measuring 30 cm in length and 5 cm in width. The angles between the arms were 120°.

Assessment criteria: The experiment was conducted over a period of 8 min, and the mice were allowed to freely explore the maze. An alternation was defined as a sequence in which a mouse entered three consecutive arms without returning to a previously visited arm. The alternation rate (%) was calculated by dividing the number of successful alternations by the total number of arm entries, as follows:

Alternation rate (%) = (Number of alternations/Total arm entries − 2) × 100.

In addition to the alternation rate, total arm entries were recorded to assess general locomotion. All behavioral data were collected and analyzed using Smart v3.0 software (Panlab, Barcelona, Spain). The time spent in each arm was also monitored to detect possible biases in the exploratory behavior.

#### 2.7.2. Barnes Maze Test

The Barnes maze test (domestically manufactured, Republic of Korea) was conducted to assess long-term cognitive memory through positive motivation. The experimental platform consisted of a circular black structure with a diameter of 92 cm and 18 holes, with all mice starting from the center of the platform at the beginning of each trial. The experiment consisted of a 3-day training phase followed by a probe phase on the 4th day. During the training phase, target holes were baited with food familiar to the mice to provide motivation. Each mouse was allowed to explore the maze freely for up to 3 min, and the trial ended early if the target zone was located quickly. In the probe phase, the latency to reach the target hole and the proportion of time spent in the target quadrant (%, TSTQ) relative to the total time were recorded and analyzed.

#### 2.7.3. Passive Avoidance Test

The passive avoidance test (Harvard Apparatus, Holliston, MA, USA) was conducted to evaluate long-term cognitive memory in mice induced by negative reinforcement. The apparatus consisted of two compartments: a light compartment and a dark compartment, separated by a closed door at the start of the experiment. Initially, the mouse was placed in the light compartment and allowed to explore freely for 1 min. After 1 min, the door connecting the two compartments was opened, allowing the mouse to move freely between the compartments for an additional 5 min.

Upon voluntary entry into the dark compartment, the door was closed 2 s later, and a mild electric shock was administered for 5 s. The test was terminated either after 5 min elapsed or when the mouse entered the dark compartment, and the latency to enter the dark compartment was recorded. This procedure was repeated for four consecutive days.

#### 2.7.4. Open Field Test

The open field test was conducted to assess locomotor activity and anxiety levels in mice. The experimental apparatus was a 45 × 45 × 40 cm (x, y, z) cubic field, with the floor divided into a central zone (22.5 × 22.5 cm) and four peripheral zones, each 11.25 × 22.5 cm. The walls were 40 cm high to prevent escape. The floor of the chamber was equipped with a grated surface that allowed detection of the animal’s movements.

Assessment criteria: Each mouse was placed in the center zone of the open field at the start of the test and allowed to explore freely for a duration of 10 min. The following parameters were measured.

Total distance traveled: The overall distance moved by the animal in centimeters.

Time spent in the central zone: This is used as an index of anxiety behavior, with increased time spent in the central zone indicating reduced anxiety.

Time spent in the peripheral zone: Increased time spent in the periphery generally reflects higher anxiety.

Velocity: The average speed of movement during the test.

#### 2.7.5. Rotarod Test

The rotarod test (Panlab, Barcelona, Spain) was conducted to evaluate the stamina and balance ability of mice. Mice were placed on a rotating rod, which started at an initial speed of 4 rpm and accelerated by 10 rpm every 10 s, reaching a maximum speed of 40 rpm. The test was performed for a maximum duration of 3 min, and the time to fall and the rotational speed of the rod at the time of falling were automatically recorded.

This experiment was conducted three times per day, and the daily average was calculated from the three trials. The procedure was repeated for a total of four consecutive days.

### 2.8. Flow Cytometry

For flow cytometry, cryopreserved MMSCs were thawed and washed with 1× BD Perm/Wash^™^ buffer (BD Biosciences, San Jose, CA, USA). Cells were incubated at 4 °C for 20 min with the following conjugated monoclonal antibodies: CD73-PE, CD90-PE, CD105-PE, CD34-PE, CD45-PE, HLA-DR-PE, Tra-1-60-FITC, and Tra-1-81-FITC (all from BD Pharmingen, CA, USA). After incubation, the cells were washed and resuspended in 1× BD Perm/Wash^™^ buffer.

For intracellular staining, cells were treated with a fixation/permeabilization solution at 4 °C for 20 min and subsequently stained with the *Oct3/4*-FITC antibody (BD Pharmingen) under the same conditions. After incubation, the cells were washed and resuspended in 1× BD Perm/Wash^™^ buffer. Data acquisition and analysis were performed using NovoExpress^®^ Software v1,3,0 (ACEA Biosciences, San Diego, CA, USA).

### 2.9. Reverse-Transcription Quantitative Polymerase Chain Reaction (RT-qPCR)

At the end of the behavioral tests experiment, the group was randomly divided for messenger RNA (mRNA) level analysis. The mice were humanely euthanized under respiratory anesthesia without pain, and brain tissues were collected.

#### 2.9.1. RNA Extraction

Next, 200 µL of chloroform was added to the homogeneously mixed tissue and solution and mixed, and the centrifuge was run at 13,000 rpm and 4 °C for 10 min. After 10 min, when the protein, DNA, and RNA layers had separated, 400 µL was taken from the RNA layer, which was the clear supernatant, and transferred to a 1.5 mL microtube; then, an equal volume of isopropanol (1:1) was added to precipitate the RNA for 10 min. After precipitation, it was centrifuged again at 13,000 rpm and 4 °C for 5 min. We checked the RNA pellet after centrifugation and carefully removed only the supernatant. After removal, we added 75% ethanol 1 mL diluted in nuclease free water (NFW) to wash away the remaining solvent. It was then centrifuged again at 10,000 rpm and 4 °C for 5 min; the supernatant was removed, and the pellet was dried. We then added 20 µL of pre-heated NFW and quantified the amount of RNA via nanodrop at 1000 ng/mL, calibrating all individuals equally.

#### 2.9.2. cDNA Synthesis

Next, the calibrated RNA dilution was added to the RNA dilution, NFW, and 2 µL of master mix according to the standard protocol provided by All-In-One 5xcDNA Master Mix (CellScript, Madison, WI, USA) for a final volume of 10 µL.

After addition, cDNA synthesis by reverse transcription was performed according to the standard protocol for cDNA synthesis provided by the T100 Thermal Cycler (BIO-R AD, Contra Costa, CA, USA). Upon completion of synthesis, 90 µL of NFW was added to dilute to 1/10.

#### 2.9.3. Reverse Transcription-Quantitative Polymerase Chain Reaction

In one well of a 96-well plate, we dispensed 19 µL of primers, SYBR green, and NFW corresponding to the transcripts to be targeted according to the standard protocol (Table 1); then. 1 µL of cDNA was added to each for a total volume of 20 µL. A PCR sealing film was placed on top of the 96 wells to prevent the contents of each well from evaporating and foreign substances from entering. The plate was loaded into the CFX96 Real-Time PCR System (BIO-RAD), and the polymerase reaction was performed according to the PCR protocol. The PCR protocol was 95 °C for 15 min, followed by 40 cycles of 95 °C for 10 s, 60 °C for 15 s, and 72 °C for 20 s.

### 2.10. Hematoxylin and Eosin Staining

The mice were humanely euthanized under respiratory anesthesia without pain, and brain tissues were collected. H&E staining was performed on mouse brain tissue sections as follows. The tissue was fixed in 4% paraformaldehyde (PFA) and dehydrated in 30% sucrose for 48 h. The dehydrated tissue was then embedded in optimal cutting temperature (OCT) compound and rapidly frozen at −20 °C to create frozen blocks. The blocks were sectioned at a thickness of 8 µm using a microtome (Thermo Scientific Shandon, Cheshire, UK) to prepare frozen tissue slides. The slides underwent rehydration by immersing them sequentially in 100%, 95%, 80%, and 70% ethanol for 2 min each, followed by washing in running water for 3 min. Hematoxylin (KP&T, Ohsong-si, Republic of Korea) staining was performed for 3 min, after which the slides were washed again in running water for 3 min. The slides were then stained with eosin (KP&T, Chungcheongbuk-do, Republic of Korea) for 30 s and dehydrated through sequential immersion in 70%, 80%, 95%, and 100% ethanol. Finally, the slides were dried, mounted with mounting medium (Fisher Chemical, Waltham, MA, USA), covered with cover slips, and observed under a microscope.

### 2.11. Immunofluorescence Staining of Brain Tissue

The mice were humanely euthanized under respiratory anesthesia without pain, and brain tissues were collected. The tissues were fixed in 4% paraformaldehyde (PFA) and dehydrated in 30% sucrose for 48 h. Subsequently, the tissues were embedded in OCT compound and rapidly frozen at −20 °C to create frozen blocks. The blocks were sectioned at a thickness of 8 µm using a microtome (Thermo Scientific Shandon, Cheshire, UK) to prepare frozen tissue slides.

For immunofluorescence (IF) staining, the slides were washed three times with PBS for 5 min each to remove residual cryoprotectant. Permeabilization and blocking were performed by incubating the slides in 0.3% Triton X-100 and 1% BSA/PBS for 40 min at room temperature. The slides were then washed again three times with PBS for 5 min each. Primary antibodies were diluted in 1% BSA/PBS according to the manufacturer’s protocol and applied to the slides, followed by incubation overnight at 4 °C. After additional PBS washes, secondary antibodies conjugated to fluorescent dyes were diluted in 1% BSA/PBS and applied to the slides for 2 h at room temperature in the dark.

Following the final PBS wash, DAPI staining was performed to visualize nuclei. DAPI was diluted 1:1000, applied to the slides, and removed by suction after 5 min. The slides were mounted using an antifade mounting medium, covered with cover slips, and observed under a fluorescence microscope. When necessary, the slides were preserved for long-term storage.

### 2.12. Statistical Analysis

All behavioral test values are expressed as the mean  ±  standard error (SEM), considering individual variability in spontaneous behaviors among mice, while RT-qPCR results are expressed as standard deviation (SD) to account for inter-sample variation. All statistical analyses were performed using GraphPad Prism 8 software (GraphPad, La Jolla, CA, USA). Statistical significance was determined using one-way ANOVA, followed by Tukey’s multiple comparisons test where applicable to identify specific group differences. The *p*-values between groups were calculated and reported to assess the significance of the observed differences.

## 3. Results

### 3.1. Phenotypic Characterization of MMSCs

**Figure 1:**
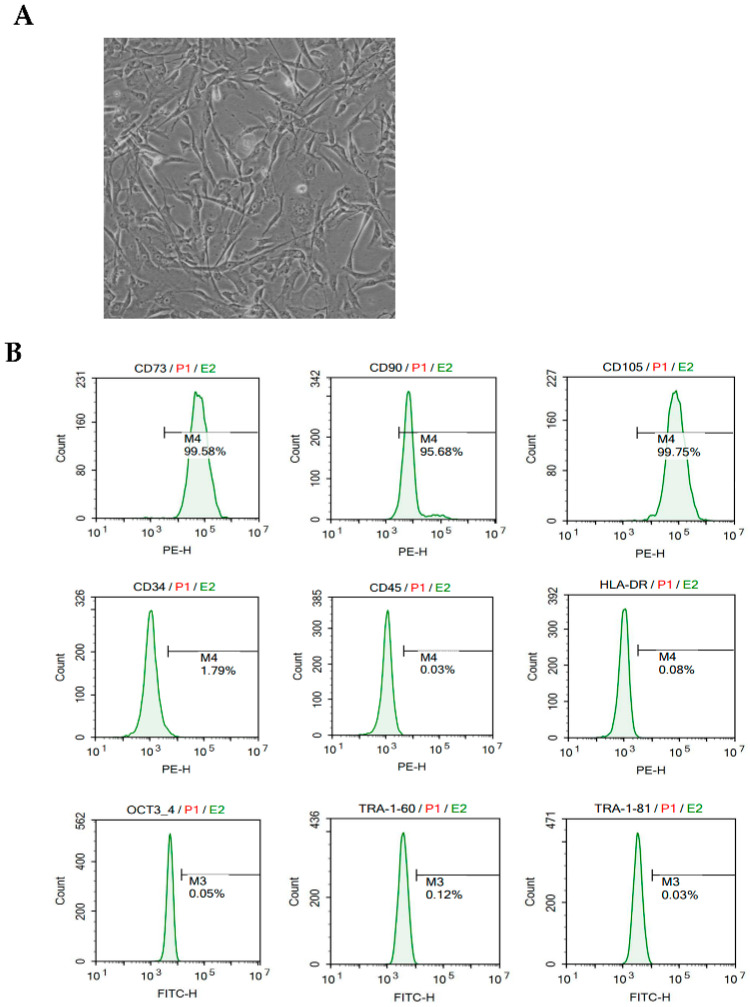
Morphological characteristics, immunophenotyping, and in vivo tracking of MMSCs. (**A**) Morphology of MMSCs: Light microscopy image at 200× magnification showing the typical fibroblast-like spindle shape of MMSCs. (**B**) Immunophenotyping results of MMSCs: MMSCs were stained with monoclonal antibodies conjugated to PE and FITC against CD73, CD90, CD105, CD34, CD45, HLA-DR, OCT3/4, TRA-1-60, and TRA-1-81. Green histograms represent the reactivity of the antibodies, and the bars indicate the ratio of antibody reactivity to the background signal. FACS analysis demonstrated positive expressions of CD73, CD90, and CD105, while CD34, CD45, HLA-DR, OCT3/4, TRA-1-60, and TRA-1-81 were negative.

MMSCs for VD treatment demonstrated a typical fibroblast-like spindle morphology with high homogeneity, as observed under light microscopy (Figure 1A). The phenotypic stability and functional suitability of hESC-MMSCs were confirmed according to the criteria for MSC characteristics and identification established by the International Society for Cellular Therapy (ISCT).

Additionally, in vivo tracking of MMSCs was performed using DiI dye staining and intracerebroventricular (ICV) injection into the mouse brain. MMSCs were labeled with DiI dye to enable visualization of their localization within the brain. DAPI staining was used to counterstain nuclei, providing structural reference for brain tissue. The results demonstrated successful delivery and retention of MMSCs in the brain, as DiI-positive signals (red) were observed in specific regions of the brain tissue. These findings indicate that MMSCs remained viable and localized at the target site after injection.

The expression of surface markers in MMSCs harvested from subculture no. 5 was analyzed using flow cytometry, showing high levels of the MSC markers CD73 (>99.58%), CD90 (>95.68%), and CD105 (>99.75%) (Figure 1B). In contrast, no expression was observed for hematopoietic lineage markers (CD34), leukocyte markers (CD45), or major histocompatibility complex markers (HLA-DR). Furthermore, hESC-specific genes (OCT4, TRA1-60, and TRA1-81) were also not expressed. These results indicate that MMSCs for VD treatment had lost pluripotency (Figure 1B). Accordingly, MMSCs exhibited the same immunophenotypic characteristics as general MSCs, meeting the ISCT criteria.

### 3.2. MMSCs Improve Working Memory in VD Mice

**Figure 2:**
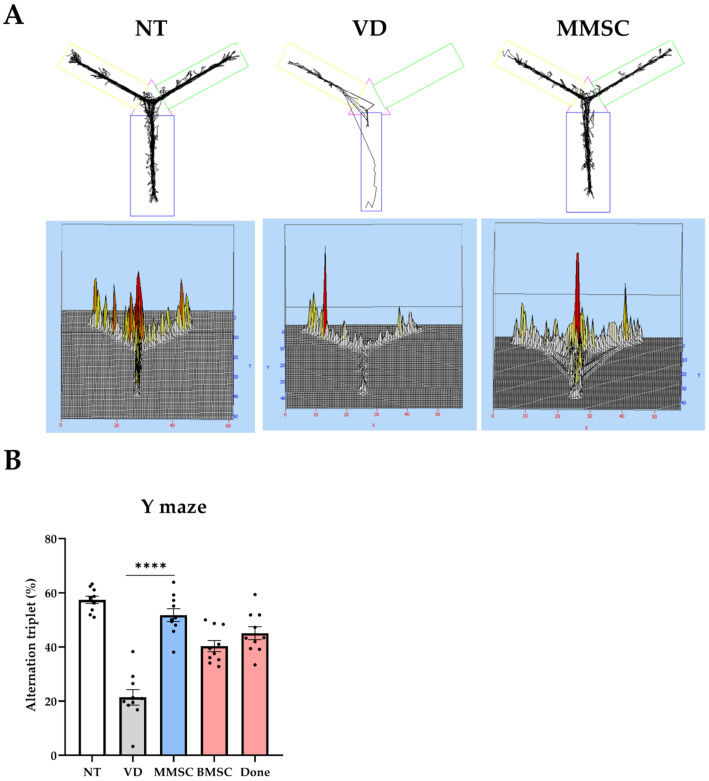
Summary of the Y-maze test. (**A**) Movement paths of each group overlapped in the Y-maze experiment for working memory measurement. (**B**) Alternation (%) in the Y-maze test. Data are presented as mean ± SEM. **** *p* < 0.001. NT: normal type; VD: vascular dementia; MMSCs: multipotent mesenchymal stem cells; BMSCs: bone marrow-derived mesenchymal stem cells; Done: donepezil.

The Y-maze test was conducted to evaluate the working memory of each group (Figure 2). MMSC demonstrated a significant improvement in working memory impaired by VD. Visualization of the movement paths of each mouse revealed that mice in the VD group exhibited repetitive movement in specific arms, whereas mice in the NT and MMSC groups crossed all three arms and achieved alternation (Figure 2A).

These results were quantified and analyzed for statistical significance (Figure 2B). The VD group recorded a lower alternation (%) compared to the NT group, while the MMSC group achieved a higher alternation (%). Following the MMSC group, the BMSC and Done groups showed relatively lower improvements.

### 3.3. MMSCs Improve Long-Term Memory in VD Mice

**Figure 3:**
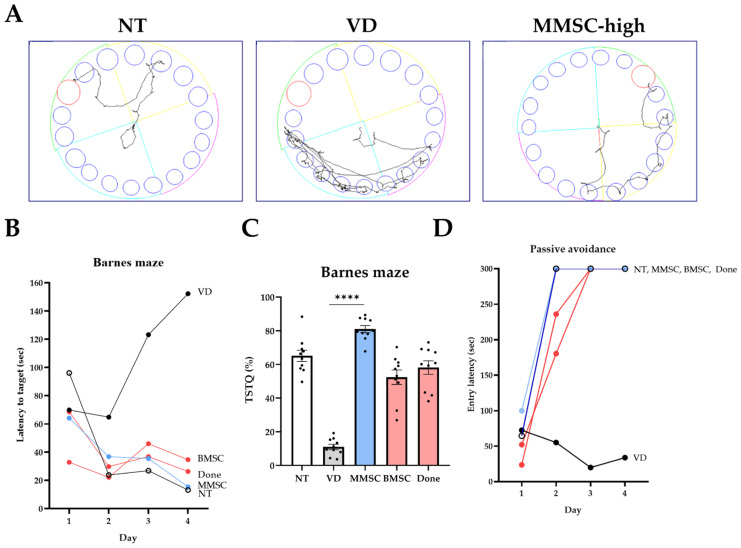
Results of the Barnes maze test and passive avoidance test. (**A**) Visualization of movement paths for each group during the Barnes maze test. (**B**) Latency to target for each group across days in the Barnes maze test. (**C**) Time spent in the target quadrant (TSTQ) as a percentage of total activity time, calculated as the time spent in the target quadrant divided by the total activity time, for each group in the Barnes maze test. Data are presented as mean ± SEM. (**D**) Entry latency (sec) into the dark compartment during the passive avoidance test across days. **** *p* < 0.0001 NT: normal type; VD: vascular dementia; MMSCs: multipotent mesenchymal stem cells; BMSCs: bone marrow-derived mesenchymal stem cells; Done: donepezil.

The Barnes maze test was conducted to evaluate long-term cognitive memory through positive reinforcement in each group. Upon analyzing the movement paths of mice in the NT, VD, and MMSC groups, the VD group demonstrated more complex movement patterns compared to WT, whereas the MMSC group exhibited efficient trajectories (Figure 3A).

When measuring the delay time to reach the target zone, the VD group exhibited significantly prolonged delay times compared to the NT group. All treatment groups showed improved results compared to the VD group, with the MMSC group demonstrating the second-best improvement after the NT group (Figure 3B).

Furthermore, when analyzing the proportion of time spent in the target quadrant (TSTQ), the VD group showed significantly lower occupancy compared to the NT group, whereas all treatment groups demonstrated higher target quadrant occupancy. Notably, the MMSC group exhibited the highest target quadrant occupancy among all groups (Figure 3C).

All groups underwent a passive avoidance test to assess their ability to respond to negative reinforcement (Figure 3D). As a result, all groups except for the VD group demonstrated significant improvements. On days 3 and 4 of the experiment, all groups except the VD group exhibited passive avoidance responses. However, on day 2, only the MMSC group showed consistent avoidance responses.

### 3.4. Effects of MMSCs Administration on Locomotor Activity and Anxiety in VD Mice

**Figure 4:**
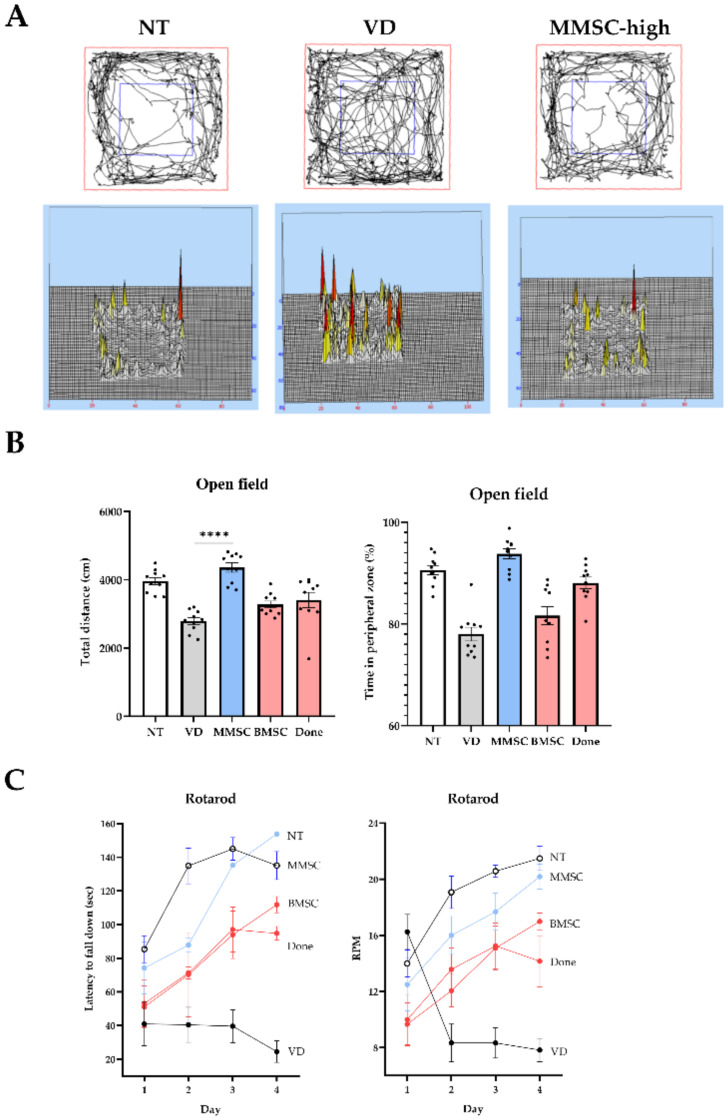
Results of the open field test and rotarod test. (**A**) Visualization of movement paths of each group in the open field chamber. (**B**) Graphs showing the total distance traveled (cm) and time spent in the peripheral zone (%) by each group during the open field test. Data are presented as mean ± SEM. (**C**) Graphs representing the latency to fall down (sec) and RPM recorded for each group during the rotarod test. **** *p* < 0.0001. NT: normal type; VD: vascular dementia; MMSCs: multipotent mesenchymal stem cells; BMSCs: bone marrow-derived mesenchymal stem cells; Done: donepezil.

An open field maze was used to assess general locomotor activity and anxiety in VD mice and to evaluate the effects of MMSC administration. When visualizing the movement paths of the NT, VD, and MMSC groups, the VD group exhibited more movement in the central zone, whereas the MMSC group showed greater movement in the peripheral zone (Figure 4A).

Measurement of total travel distance for each group revealed that the VD and Done groups exhibited significantly reduced total distances compared to the NT group. In contrast, both the MMSC and BMSC groups showed improved travel distances, with the MMSC group demonstrating a greater improvement (Figure 4B). Additionally, analysis of time spent in the peripheral zone indicated that the VD, BMSC, and Done groups spent less time in the peripheral zone compared to the NT group, whereas the MMSC group spent a significantly longer time in this zone.

The rotarod test was conducted to assess balance ability and motor learning in VD mice and to evaluate the efficacy of MMSC treatment. When measuring the delay time before falling as the rod speed increased, the VD group fell significantly faster than the NT group and at lower RPMs. However, all treatment groups showed improvements, with the MMSC group demonstrating the most significant improvement (Figure 4C).

### 3.5. Effects of MMSCs Administration on Gene Expression and Histological Recovery in VD Mice

To evaluate the effects of MMSC administration on VD recovery, mRNA was extracted from the brains of mice in each group, followed by reverse transcription and RT-qPCR to measure gene expression levels.

**Figure 5:**
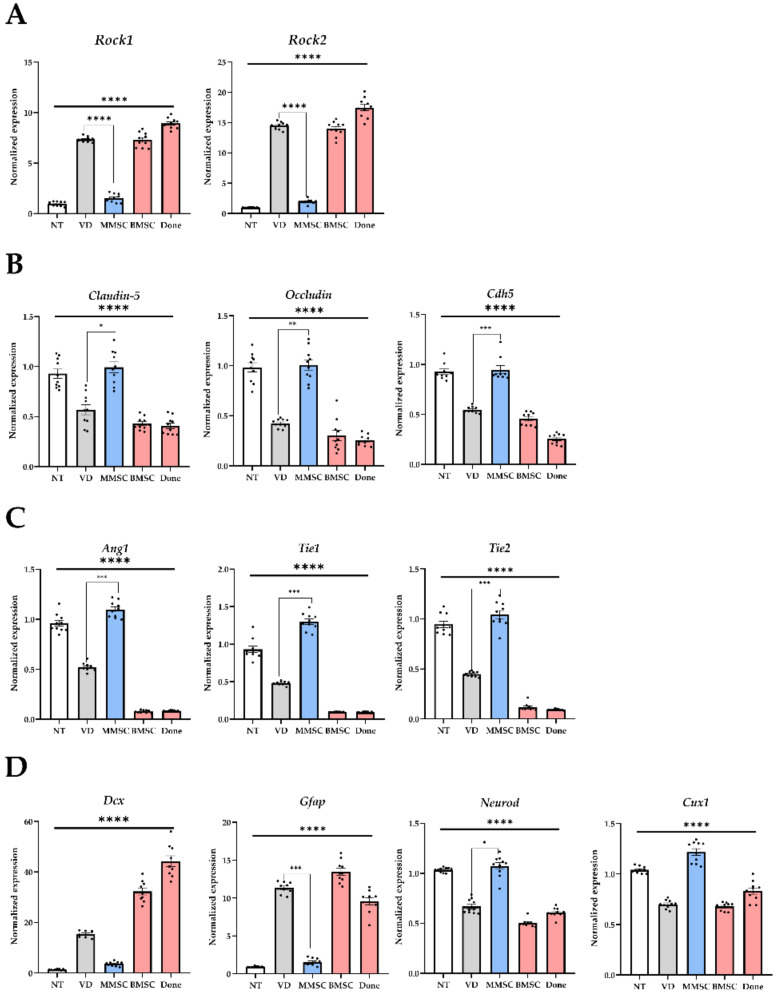
mRNA expression levels analyzed from brain tissues of mice in each group. (**A**) Genes associated with the ROCK pathway, (**B**) genes related to the BBB, (**C**) genes involved in angiogenesis, and (**D**) genes associated with neuronal and glial cells. All graphs are presented as mean ± standard deviation (SD). * *p* < 0.05; ** *p* < 0.01; *** *p* < 0.001; **** *p* < 0.0001. NT: normal type; VD: vascular dementia; MMSCs: multipotent mesenchymal stem cells; BMSCs: bone marrow-derived mesenchymal stem cells; Done: donepezil.

The expression of *Rock1* and *Rock2*, associated with the Rock signaling pathway, was analyzed. In the VD group, the expression of *Rock1* and *Rock2* was significantly elevated compared to the NT group. MMSC administration reduced the expression levels of *Rock1* and *Rock2* to those of the NT group, whereas no reduction was observed in the BMSC and Donepezil groups (Figure 5A).

The expression levels of genes related to the TJ and adhesion junctions (AJ) of vascular endothelial cells, key components of the BBB, were also assessed. The mRNA levels of *Claudin-5*, *Occludin*, and *Cdh5* were significantly reduced in the VD group compared to the NT group. MMSC administration restored the expression of these genes to levels comparable to the NT group, while BMSC and Donepezil groups failed to show significant recovery (Figure 5B). These results suggest that VD impairs the integrity of the BBB by reducing TJ and AJ protein expression.

The expression levels of *Ang1*, *Tie1*, and *Tie2*, which are involved in angiogenesis, were significantly lower in the VD group compared to the NT group. MMSC administration significantly increased their expression and restored them to levels similar to the NT group, while BMSC and Donepezil groups showed no significant recovery (Figure 5C). These findings indicate that MMSC administration can mitigate vascular dysfunction caused by reduced angiogenic gene expression in VD mice.

The expression of genes associated with neuroregeneration and glial cells, including *Dcx*, *Gfap*, *Neurod*, and *Cux1*, was also analyzed. Astrocyte-specific markers *Dcx* and *Gfap* were significantly upregulated in the VD group, whereas MMSC administration reduced their expression levels. In contrast, neuron-specific markers *Neurod* and *Cux1* were significantly downregulated in the VD group, but MMSC administration restored their expression to levels comparable to the NT group. No such recovery was observed in the BMSC and Donepezil groups (Figure 5D).

**Figure 6:**
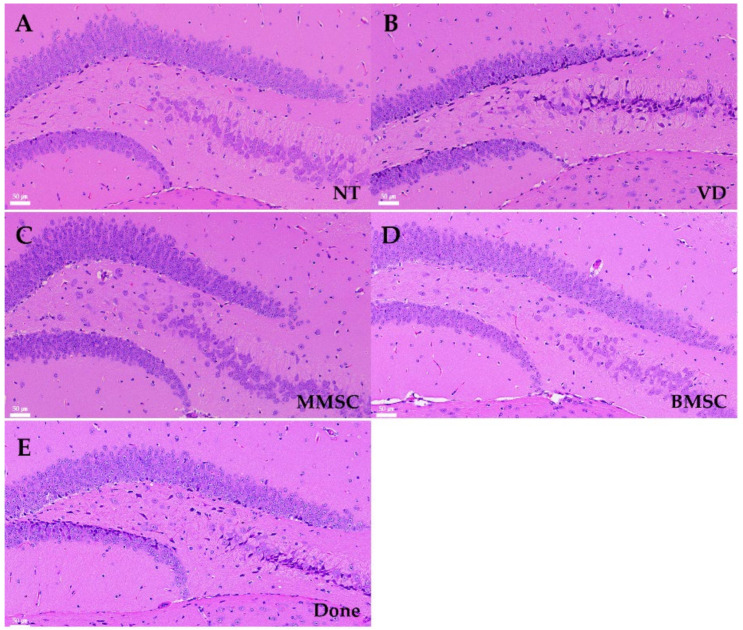
H&E staining of brain sections from each group of mice, observed at 200× magnification. The images show the CA3 and dentate gyrus (DG) regions of the hippocampus. Nuclei were stained purple, and cytoplasm was stained pink. (**A**) NT group, (**B**) VD group, (**C**) MMSC group, (**D**) BMSC group, and (**E**) Done group. NT: normal type; VD: vascular dementia; MMSC: multipotent mesenchymal stem cell; BMSC: bone marrow-derived mesenchymal stem cell; Done: donepezil. Representative images from n = 5 mice, with 4–5 sections per mouse analyzed.

Hematoxylin and eosin (H&E) staining was performed to assess neuronal arrangement and cell death in the hippocampal tissue of VD mice (Figure 6). Compared to the NT group, the VD group exhibited irregular neuronal arrangement and increased cell death in the CA3 region of the hippocampus (Figure 6A,B). Both MMSC and BMSC treatments improved neuronal survival in the hippocampus; however, the BMSC-treated group showed uneven spacing between nuclei in the CA3 region (Figure 6C,D). In contrast, the Done group did not show significant improvements in cell survival (Figure 6E).

The cerebral cortex is a collection of neurons located on the brain’s surface that is responsible for memory processing. In the mice from the VD group, there were neuronal cell arrangement irregularities (Figure 6A–E). These results were consistent with the observed decrease in the mRNA expression of the cortical marker Cux1, suggesting that the damage to the cortex and hippocampus increased due to decreased cerebral blood flow in the mice from the VD group.

**Figure 7:**
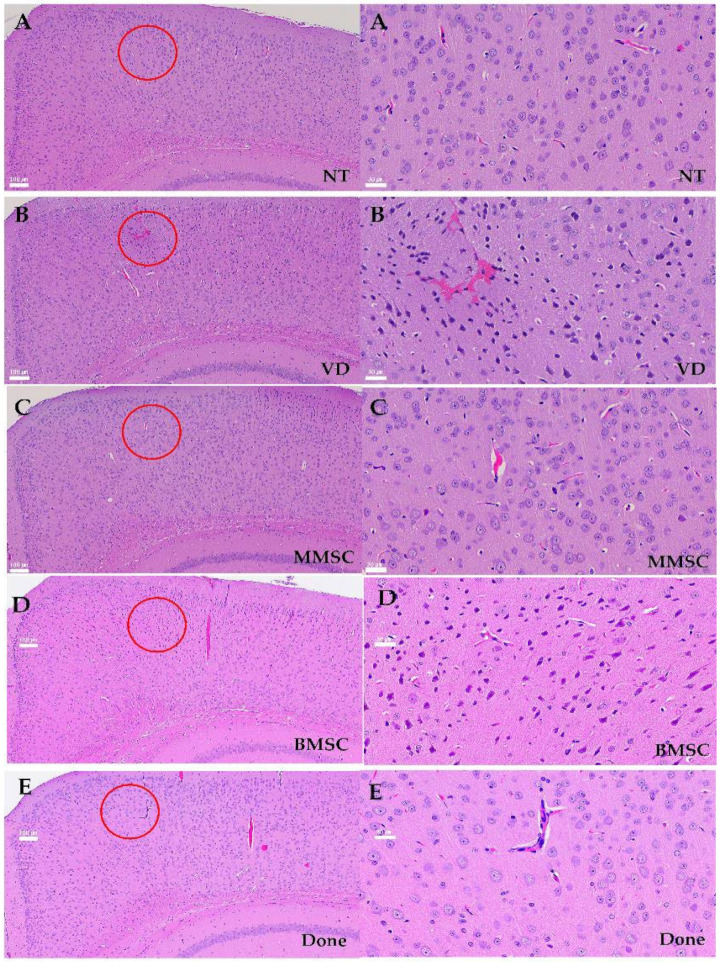
The morphology and structure of neurons in cortex of mouses, HE×100 (**Left A–E**) or 400 (**Right A–E**). (**A**) NT group, (**B**) BCAS group, (**C**) MMSC (**D**) BMSC group, and (**E**) Done group. Irregular neuron arrangement, pyknotic and dark nuclei, and a large number of vacuoles appeared in the VD group, and normal neuron arrangement and nuclei were observed in the MMSC group. (Red circle) However, nuclear condensation similar to VD group was observed in the BMSC group, and a large number of vacuole were observed in the Done group. Representative images from n = 5 mice, with 4–5 sections per mouse analyzed.

The cerebral cortex is a collection of neurons located on the brain surface, and the hippocampus is responsible for memory processing. In mice from the VD group, neuronal cell arrangement irregularities such as disrupted laminar organization, uneven spacing between neurons, and loss of tightly packed neuronal layers were observed (Figure 7A–E, left), along with pyknotic and dark nuclei in the stratum corneum (CA3) region of the hippocampus (Figure 7A–E, right). These results were identical to the decrease in the mRNA expression of the cortical marker Cux1, suggesting that cortical and hippocampal damage increased due to decreased cerebral blood flow in mice from the VD group.

## 4. Discussion

VD, caused by reduced cerebral blood flow, leads to cognitive and behavioral impairments, making it the second most common type of dementia after Alzheimer’s disease [9]. Despite its prevalence, no FDA-approved treatments are currently available for VD. With the growing prevalence of VD driven by an aging population, the development of novel therapeutic strategies to address its underlying pathophysiology is urgently needed.

This study demonstrated that MMSCs derived from human embryonic stem cells significantly improved cognitive and motor impairments in a VD mouse model. Compared to adult stem cell-derived MSCs, MMSCs exhibited superior proliferation capacity and functional efficacy. MMSCs ameliorated pathological changes associated with VD by stabilizing the BBB, promoting angiogenesis, and supporting neuronal protection and regeneration, particularly in the hippocampal region. Notably, MMSCs inhibited the Rho-associated kinase (Rock) pathway, a critical regulator of BBB integrity and inflammatory responses, thereby restoring TJ and AJ protein expression and maintaining vascular stability. These findings highlight the potential of MMSCs to play a pivotal role in VD therapy. Furthermore, MMSCs exert their therapeutic effects through multiple cellular mechanisms, including modulation of inflammatory responses, enhancement of neuroprotection, and promotion of angiogenesis. MMSCs increase the secretion of anti-inflammatory cytokines (IL-10 and TGF-β) while reducing pro-inflammatory mediators (TNF-α and IL-1β), thereby alleviating neuroinflammation. Additionally, MMSCs enhance neuronal survival and synaptic plasticity by secreting BDNF, NGF, and GDNF, which are crucial for cognitive function recovery. By activating the PI3K/Akt and Wnt/β-catenin pathways, MMSCs support neuronal regeneration and vascular remodeling, further reinforcing their therapeutic potential. Moreover, exosomes derived from MMSCs contain pro-repair miRNAs (e.g., miR-124 and miR-21) that facilitate neuroprotection and BBB stability.

In comparison to previous studies, MMSCs demonstrated superior efficacy in restoring BBB integrity and improving vascular function relative to adult MSC-based therapies. While human umbilical cord-derived MSCs (hUC-MSCs) have shown limited effects in previous studies, this research underscores the enhanced therapeutic potential of MMSCs, driven by their robust growth and scalability. The superior therapeutic efficacy of MMSCs compared to adult MSCs may be attributed to their enhanced secretion of pro-angiogenic and neurotrophic factors as well as their ability to modulate key neuroinflammatory and apoptotic pathways. Unlike adult MSCs, MMSCs exhibit a greater capacity for Rock1/2 inhibition, which plays a crucial role in maintaining vascular stability and reducing neuroinflammation. Additionally, MMSCs secrete higher levels of VEGF, BDNF, and miRNA-loaded exosomes, further supporting neurovascular recovery in VD models. Furthermore, the ability of MMSCs to secrete pro-angiogenic and immunomodulatory factors establishes them as a compelling alternative to existing cell-based therapies. By suppressing Rock1/2 expression, MMSCs reduced neuroinflammation and protected against neuronal apoptosis, offering a novel mechanism for VD treatment.

An essential aspect of preclinical research is evaluating potential risks and ensuring treatment safety. While MMSCs exhibited promising therapeutic effects, potential risks such as abnormal proliferation, immune rejection, and vascular complications must be carefully assessed. Excessive angiogenesis could lead to microvascular dysfunction, and unintended immune responses may reduce treatment efficacy. Future studies should investigate optimal dosing strategies and long-term effects to mitigate these risks. Additionally, comprehensive behavioral assessments beyond cognitive improvement are necessary to rule out any unintended neurophysiological alterations.

However, several limitations of this study should be acknowledged. First, the long-term safety and potential tumorigenicity of MMSCs were not evaluated. Although previous studies have reported that MMSCs do not induce tumor formation up to 12 months after transplantation, further validation across diverse models is required. Second, the durability of cognitive and motor improvements following MMSC treatment remains unclear. Future studies should investigate the effects of repeated or prolonged administration of MMSCs and their efficacy in chronic VD models. Expanding these investigations to include other neurodegenerative disorders could further elucidate the therapeutic scope of MMSCs.

Finally, clinical translation of MMSCs requires the standardization of production processes and quality control measures to ensure scalability and reproducibility. Assessing the cost effectiveness of MMSC-based therapies compared to conventional pharmacological treatments will also be critical for their broader application. By targeting neuroinflammatory, neurovascular, and neurodegenerative mechanisms, MMSCs offer a promising therapeutic strategy for VD, with potential applications extending to other neurodegenerative diseases.

## 5. Conclusions

This study demonstrated the therapeutic potential of MMSCs in addressing the core pathophysiological mechanisms of VD. MMSCs effectively stabilized the BBB, promoted angiogenesis, suppressed inflammation, and supported neuronal protection, leading to significant improvements in cognitive and motor functions. These findings suggest that MMSCs represent a novel and effective cell-based therapeutic strategy for VD and other neurodegenerative diseases. Future research should focus on evaluating the long-term safety, efficacy, and clinical applicability of MMSCs to advance their development as a viable therapeutic option. In this study, we utilized the bilateral common carotid artery stenosis (BCAS) model to investigate the impact of chronic cerebral hypoperfusion on vascular dementia pathology and cognitive function. The BCAS model effectively induces small vessel disease-related white matter lesions and cognitive impairment, making it a valuable tool for studying the mechanisms of vascular dementia. In particular, it allows for the examination of neuronal damage, microvascular changes, and inflammatory responses under chronic hypoperfusion conditions.

However, this model has several limitations. First, BCAS does not fully replicate the diverse pathological mechanisms of vascular dementia, particularly those involving multiple infarctions or direct cortical damage. Second, individual variations in the development of the Willis circle can lead to differences in the degree of cerebral hypoperfusion, potentially affecting the reproducibility of results. Third, BCAS primarily induces white matter-associated cognitive deficits, which may not fully reflect hippocampus-dependent memory impairments commonly observed in vascular dementia. Lastly, this model does not account for metabolic risk factors such as hypertension and diabetes, which are frequently associated with human vascular dementia.

To address these limitations, future studies should consider combining BCAS with hypertension-induced models, incorporating multiple microinfarct models, and utilizing genetically modified mice to enhance the translational relevance of this model. Such integrative approaches will provide a more comprehensive understanding of vascular dementia pathology and contribute to the development of effective therapeutic strategies.

## Figures and Tables

**Table 1:** Nucleotide sequences of primers used in RT-qPCR for mouse host.

Gene Name	Primer Type	Sequence (5′->3′)	Accession Number
*Rock1*	Forward	5′-AGA ATG AGG ACA GCA GAA GAA G-3′	NM_009071.2
	Reverse	5′-GGT CCG TTC GGT ACT GAT ATT C-3′	
*Rock2*	Forward	5′-GGA GGT ACG ACT TGG AAG AAA T-3′	NM_009072.2
	Reverse	5′-GCT GCC GTC TCT CTT ATG TTA T-3′	
*Claudin-5*	Forward	5′-CCT TCC TGG ACC ACA ACA TC-3′	NM_013805.4
	Reverse	5′-CGC CAG CAC AGA TTC ATA CA-3′	
*Occludin*	Forward	5′-GAG CTT ACA GGC AGA ACT AGA C-3′	NM_001360536.1
	Reverse	5′-CAG CCA TGT ACT CTT CAC TCT C-3′	
*Cdh5*	Forward	5′-CAG TGA CAG AGG CCA ATT CT-3′	NM_009868.4
	Reverse	5′-GCC TCC ACA GTC AGG TTA TAC-3′	
*Ang1*	Forward	5′-GTT CTA CAC TGC GGG ACA AA-3′	NM_001286062.1
	Reverse	5′-ATC ATG GTG GTG GAA CGT AAG-3′	
*Tie1*	Forward	5′-CTG AAG CCA AAG ACA GGA TAC A-3′	NM_011587.2
	Reverse	5′-GTC AGT AGT CAT AAG GGC AGA AG-3′	
*Tie2*	Forward	5′-TTT GCC CTC CTG GGT TTA TG-3′	NM_001290549.1
	Reverse	5′-CTT CTG GTC CAC TAC ACC TTT C-3′	
*Dcx*	Forward	5′-AGG GAG TGC GCT ACA TTT ATA C-3′	NM_001110222.1
	Reverse	5′-GTT GTC TGA GGA GCA GAC ATA G-3′	
*Gfap*	Forward	5′-AAC AAC CTG GCT GCG TAT AG-3′	NM_001131020.1
	Reverse	5′-TCT CGA ACT TCC TCC TCA TAG AT-3′	
*Neurod*	Forward	5′-TGA CCA AAT CAT ACA GCG AGA G-3′	NM_010894.3
	Reverse	5′-TTC TTG TCT GCC TCG TGT TC-3′	
*Cux1*	Forward	5′-CTT AGT CTG AAA GGA CGG GAA C-3′	NM_001291233.2
	Reverse	5′-CTT CTC CAT CCG CTT CAT ATC C-3′	

## Data Availability

The data generated in this study have been submitted exclusively to the journal’s submission system and are not publicly available. Access to the data will be granted upon request from the editorial board.
